# Abrupt versus gradual smoking cessation with pre-cessation varenicline therapy for Chinese treatment-seeking smokers: A retrospective, observational, cohort study

**DOI:** 10.18332/tid/145993

**Published:** 2022-03-14

**Authors:** Ning Zhu, Shanhong Lin, Luyan Dai, Hang Yu, Ning Xu, Weina Huang, Xiaopin Yu

**Affiliations:** 1 Department of Respiratory and Critical Care Medicine, Ningbo First Hospital, Ningbo, China; 2 Department of Ultrasound, Ningbo First Hospital, Ningbo, China; 3 Department of Prevention and Health Care, Ningbo First Hospital, Ningbo, China

**Keywords:** varenicline, efficacy of smoking cessation therapies, abrupt cessation, gradual cessation, adverse events

## Abstract

**INTRODUCTION:**

This study aimed to explore the efficacy of abrupt and gradual smoking cessation with pre-cessation varenicline therapy.

**METHODS:**

A total of 278 smokers who experienced moderate-to-severe nicotine dependence and visited a Chinese smoking cessation outpatient clinic from March 2017 to February 2021 were enrolled. This was a retrospective, observational, cohort study. Participants were divided into two groups by the cessation strategy they received: the abrupt cessation group (n=139, tobacco was not controlled during the first 3 weeks before the target cessation date and smoking was entirely discontinued on the 22nd day) and the gradual cessation group (n=139, tobacco was gradually reduced in the first 3 weeks before the target cessation date and smoking was discontinued on the 22nd day). The abstinence rates were compared between groups (7-day point prevalence abstinence rates at 1, 3 and 6 months post-treatment; and 1-month and 3-month continuous abstinence rates of 6-month follow-up). Possible factors that influence efficacy, reasons for smoking cessation failure, and associated adverse events were also analyzed.

**RESULTS:**

No significant difference in the 7-day point prevalence abstinence rates at 1, 3 and 6 months post-treatment was observed between the groups (p>0.05). The 1-month continuous abstinence rate of the gradual cessation group was higher than that of the abrupt cessation group (51.1% vs 31.7%; χ^2^=10.812, p=0.001). The 3-month continuous abstinence rate of the gradual cessation group was also higher than that of the abrupt cessation group (42.4% vs 27.3%; χ^2^=6.983, p=0.008). Abrupt cessation was a risk factor for successful smoking cessation than gradual cessation (AOR=2.39; 95% CI: 1.15–3.85, p=0.013),the motivation of ‘prevention and treatment of own diseases’ reduced the risk of incomplete abstinence (AOR=0.87; 95% CI: 0.38–0.99, p=0.049). The incidence of adverse events was higher in the abrupt cessation group than in the gradual cessation group. The incidence rates of nausea and insomnia were statistically significant differences.

**CONCLUSIONS:**

Compared with abrupt cessation, gradual smoking cessation with pre-cessation varenicline therapy produced higher abstinence rates and relatively milder withdrawal symptoms.

## INTRODUCTION

Studies have shown that around half of the smokers who attempt to quit smoking choose to gradually reduce tobacco use before achieving complete abstinence^1,2^. However, current guidelines for smoking cessation recommend abrupt quitting interventions for smokers who make active quit attempts^3^. In clinical practice, some smokers cannot quit smoking abruptly due to the high addiction to tobacco and the lack of psychological preparedness for smoking cessation, but they can achieve ultimate abstinence by gradually reducing tobacco use^4^. Gradual cessation means gradually reducing the number of cigarettes smoked as planned over a predetermined period of time, which allows time for smokers to adapt both physiologically and psychologically. Results of relevant studies indicated that^5-7^ excessively long course of smoking cessation treatment tended to lower the smokers’ motivation to cease smoking, while excessively short course of treatment tended to dent the smokers’ confidence in cessation success due to difficulty in achieving abstinence. Gradual cessation allows smokers who lack confidence in cessation success to make quit attempts, but the predetermined duration of the gradual cessation therapy and whether gradual cessation is as effective as abrupt cessation in achieving abstinence are yet to be determined. Moreover, most previous studies^8^ focused on the comparison of abstinence rates between the two cessation strategies in nicotine replacement therapy (NRT)-aided smoking cessation and further research on the abstinence rates produced by the two cessation methods in varenicline tartrate-aided smoking cessation is needed. Therefore, in this retrospective study, we enrolled smokers who underwent varenicline-aided cessation treatment, categorized the enrolled cases into the gradual cessation group and the abrupt cessation group, and defined the cessation treatment course as 3 weeks^6,7^ based on relevant studies and clinical practice. The abstinence rates of the two groups, relevant contributing factors, reasons for smoking cessation failure, and the adverse events were discussed.

## METHODS

### Study participants

**Figure 1:**
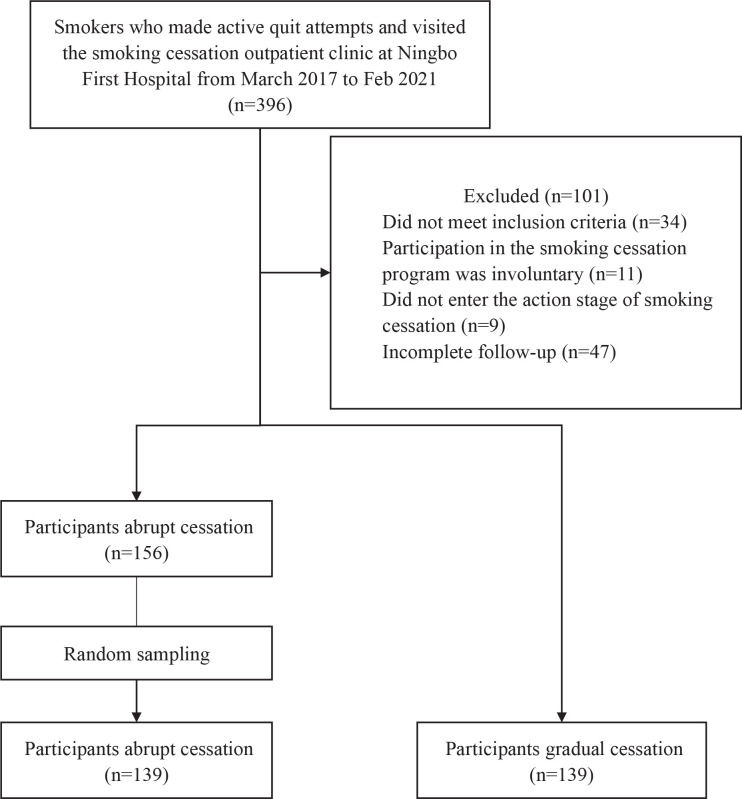
Study flow diagram

A total of 278 smokers who made active quit attempts and visited the smoking cessation outpatient clinic at Ningbo First Hospital from March 2017 to February 2021 were enrolled in this retrospective, observational, cohort study. The enrolled cases were divided into the gradual cessation group (n=139) and the abrupt cessation group (n=139) according to the cessation strategy they received. The study flow diagram is shown in Figure 1.

The inclusion criteria were: 1) smokers who experienced moderate-to-severe nicotine dependence, 2) smokers who made voluntary quit attempts and underwent drug interventions (persistent or cumulative cigarette use ≥2 years); daily cigarette use (≥5 cigarettes/day) prior to the outpatient visit and nicotine dependence score (>3)^8^ at the outpatient visit; and 3) smokers who entered the action stage of smoking cessation, accepted the target quit dates, and could complete the follow-up visits on time. Those who self-reported continuing to smoke and the actual detected concentration of carbon monoxide in exhaled breath was greater than or equal to the base value, were considered to have failed to enter the action stage of smoking cessation.

The exclusion criteria were: 1) smokers whose participation in the smoking cessation program was involuntary, 2) incomplete follow-up; and 3) smokers who did not enter the action stage of smoking cessation.

### Data collection

Doctors at the smoking cessation outpatient clinic collected the baseline data for the smokers at their first outpatient visit, including demographic characteristics, such as gender, age, age at smoking onset, daily cigarette use, the number of years smoked, Fagerström test for nicotine dependence (FTND) score^9^, marital status, employment status, level of education, underlying diseases, motivation to quit, and the number of past quit attempts. Motivation to quit smoking was measured using single item scales. The motivation was assessed on a 0–10 point scale by the question: ‘How motivated are you to quit smoking?’, with 0 not at all motivated and 10 extremely motivated to quit smoking. A similar 0–10 response scale has been successfully used to assess motivation and confidence for smoking behavior change in prior studies^10,11^. The Micro II smokerlyzer was used to detect the concentration of carbon monoxide in the exhaled breath (FeCO) of smokers (evaluation criteria: 0–6 mild; 7–10 moderate; 11–20 severe; and >20 extremely severe). All subjects who experienced ultimate failure in smoking cessation were asked about the reasons for their failure: inability to overcome tobacco addiction, the need to relieve work stress by smoking, social influence of other smokers, lack of psychological preparedness and perseverance for smoking cessation, stress and anxiety over the course of smoking cessation, depression and unhappiness over the course of smoking cessation, and other reasons such as lack of confidence in cessation success and fear of post-abstinence weight gain. Associated adverse events that occurred over the course of smoking cessation (24 weeks) were followed up.

### Exposure factors

The subjects were categorized as the gradual cessation group and the abrupt cessation group, and the 22nd day after enrollment was set as the target quit date.

In the gradual cessation group, cigarette use was reduced to 3/4 of baseline smoking in the first week, 1/2 of baseline smoking in the second week, and 1/4 of baseline smoking in the third week. Moreover, smoking was entirely discontinued since the 22nd day after enrollment, and drug-facilitated smoking cessation treatment was delivered simultaneously since the first day.

In the abrupt cessation group, the specific amount of smoking was not defined for the first 3 weeks. Smoking was entirely discontinued since the 22nd day after enrollment, and drug-facilitated smoking cessation treatment was also delivered since the first day.

The two groups underwent drug therapy after the target quit date and the course of the drug therapy was jointly decided by the smokers and the doctors. Varenicline tartrate (Pfizer from US) was used in the drug-facilitated smoking cessation treatment. The dosages were as follows: Days 1 to 3, 0.5 mg once daily; Days 4 to 7, 0.5 mg twice daily; and starting from Day 8, 1 mg twice daily. Smoking cessation tips and psychological guidance were also offered over the course of the therapy^12-15^.

The enrolled smokers who completed their first visit at the smoking cessation outpatient clinic were followed up. The follow-up methods included outpatient follow-up and phone or WeChat (the most widely used communication app in China) follow-up. At least one follow-up visit at the smoking cessation outpatient clinic was made before the target quit date. Three outpatient follow-up visits were made at 1, 3 and 6 months after the target quit date, and phone follow-up was performed once per month. As for WeChat follow-up, the smoking cessation physician sent invitation codes or QR codes to the patients’ phones at their first outpatient visit. After confirmation by the smoker, he/she would join a WeChat group dedicated for smoking cessation. In the first 4 weeks, smoking cessation tips were delivered to this WeChat group twice per week. In weeks 5–24, smoking cessation tips were delivered once per week. If the patient encountered problems over the course of smoking cessation, he/she could communicate these problems anytime via WeChat where a smoking cessation physician would provide solutions. The following information was collected during follow-up visits: self-reported health status, progress in smoking cessation, the number of cigarettes smoked daily, the use of medications, the detected concentration of carbon monoxide in exhaled breath, reasons for failure in smoking cessation, relevant adverse events, and other relevant information.

### The evaluation index

Main evaluation index (continuous abstinence rates): self-reported smoking cessation for ≥1 month (1-month continuous abstinence rate) or ≥3 months (3-month continuous abstinence rate) at the 6-month follow-up, and a detected concentration of carbon monoxide in exhaled breath of ≤6 ppm.

Secondary evaluation index: 1) point prevalence abstinence rates: self-reported recent tobacco abstinence for ≥7 days at follow-up at 1, 3 and 6 months, and a detected concentration of carbon monoxide in exhaled breath of ≤6 ppm; 2) relevant adverse events over the course of smoking cessation (24 weeks); and 3) incomplete abstinence, defined as any quit attempt that ended in failure (i.e. resumed smoking) identified at a given follow-up assessment.

### Statistical analysis

We estimated a gradual cessation group success rate of about 50%, cessation can increase the success rate by 20%, using a two-sided α of 5%, and participants per group to have 90% power to detect this effect.

SPSS Statistics 21.0 was used, with age, age at smoking onset, the number of cigarettes smoked daily, the number of years smoked, and FTND score expressed as mean and standard deviation. The analysis of variance and chi-squared (χ^2^) tests were performed to compare gender, marital status, employment status, level of education, motivation to quit, the number of past quit attempts, concomitant underlying diseases, FeCO at the first outpatient visit, and abstinence rates and adverse events between the two groups. Incomplete abstinence was used as the dependent variable, with all the factors shown in Table 1 and the methods of smoking cessation (abrupt and gradual) as the independent variables. Multiple logistic regression analysis was used to determine the relevant factors that affect the incomplete abstinence. A p<0.05 was considered statistically significant.

**Table 1:** Basic characteristics and demographic data of varenicline-aided gradual and abrupt smoking cessation (N=278)

*Characteristics*	*Abrupt group (n=139) n (%)*	*Gradual group (n=139) n (%)*	*Statistic*	*p*
**Age** (years), mean ± SD	52.4 ± 10.5	51.1 ± 11.3	F=0.487	0.472
**Age started smoking** (years), mean ± SD	23.1 ± 11.8	22.8 ± 13.2	F=0.477	0.483
**Cigarettes per day,** mean ± SD	22.2 ± 13.2	21.8 ± 10.7	F=0.164	0.671
**Smoking duration** (years), mean ± SD	25.1 ± 10.4	23.4 ± 11.2	F=0.534	0.476
**FTND score,** mean ± SD	4.73 ± 2.3	4.8 ± 2.2	F=0.231	0.649
**Gender**				
Male	133 (49.3)	137 (50.7)		
Female	6 (75.0)	2 (25.0)	χ^2^=2.059	0.151
**Marital status**				
Married	114 (51.1)	109 (48.9)		
Other	25 (45.5)	30 (54.5)	χ^2^=0.567	0.452
**Educational level**				
Primary (0–9 years)	71 (52.6)	64 (47.4)		
Secondary (9–12 years)	32 (43.8)	41 (56.2)		
Higher (>12 years)	36 (51.4)	34 (48.6)	χ^2^=1.530	0.465
**Employment status**				
Currently employed	103 (49.0)	107 (51.0)		
Student/unemployed/retired/				
other	36 (52.9)	32 (47.1)	χ^2^=0.311	0.577
**Prior attempts to quit smoking**				
Yes	88 (47.6)	97 (52.4)		
No	51 (54.8)	42 (45.2)	χ^2^=1.309	0.253
**Quitting motivation**				
Prevention and treatment of own diseases	66 (46.2)	77 (53.8)		
Mobilization of others	54 (57.4)	40 (42.6)		
Other	19 (46.3)	22 (53.7)	χ^2^=3.151	0.207
**Comorbidities**				
None	26 (45.6)	31 (54.4)		
Respiratory	76 (50.7)	74 (49.3)		
Non-respiratory	37 (52.1)	34 (47.9)	χ^2^=0.592	0.744
**Exhaled CO at first visit** (ppm)				
0–6	22 (53.7)	19 (46.3)		
7–10	34 (47.2)	38 (52.8)		
11–20	63 (47.0)	71 (53.0)		
>20	20 (64.5)	11 (35.5)	χ^2^=3.532	0.317

## RESULTS

### Characteristics of smokers

A total of 278 smokers were enrolled, including 270 males with mean age of 51.2±13.7 years and 8 female cases with mean age of 41.3±8.7 years. Overall, 57 cases (20.5%) were previously healthy, and 150 cases (54%) had concomitant respiratory diseases; 71 cases (25.5%) had concomitant diseases in other systems, including cardiovascular and cerebrovascular diseases (31 cases), digestive system disorders (12 cases), pharyngolaryngitis (4 cases), diabetes mellitus (9 cases), mental disorders (1 case), renal cyst (7 cases), renal cancer (2 cases), connective tissue diseases (2 cases), iron-deficiency anemia (1 case), dermatosis (1 case), and hyperuricemia (1 case). Moreover, 143 cases (51.4%) decided to quit smoking in order to protect their health, 94 cases (33.8%) were persuaded by others to participate in the smoking cessation program, and 41 cases (14.7%) decided to quit smoking owing to other reasons (e.g. pre-pregnancy preparation, collective smoking cessation, etc.).

No significant difference was observed in terms of age, number of cigarettes smoked daily, FTND score, gender, employment status, motivation to quit, level of education, underlying diseases, and FeCO at the first outpatient visit between the abrupt cessation group and the gradual cessation group (p>0.05 for all indicators) (Table 1).

### Comparison of abstinence rates between the two groups

The 7-day point prevalence abstinence rates of the gradual cessation group at 1, 3 and 6 months post-treatment were 54.0%, 41.7%, and 53.2%, respectively, indicating no statistically significant difference when compared with the abrupt cessation group (50.4%, 45.3%, and 41.7%; χ^2^=0.360, 0.366, and 3.693, p>0.05). The results suggest that gradual cessation produced short-term abstinence rates close to that of abrupt cessation. The 1-month continuous abstinence rate (51.1%) of the gradual cessation group was significantly higher than that of the abrupt cessation group (31.7%; χ^2^=10.812, p=0.001), whereas the 3-month continuous abstinence rate of the gradual cessation group (42.4%) was still higher than that of the abrupt cessation group (27.3%; χ^2^=6.983, p=0.008). The results suggested that the gradual cessation group produced higher long-term abstinence rates than those of the abrupt cessation group (Table 2).

**Table 2:** Comparison of abstinence rates between varenicline-aided gradual and abrupt smoking cessation (N=278)

*Outcome measures*	*Abrupt group (n=139) n (%)*	*Gradual group (n=139) n (%)*	*χ^2^*	*p*
**7- day point prevalence abstinence rates**				
Follow-up at 1 month	70 (50.4)	75 (54.0)	0.360	0.548
Follow-up at 3 months	63 (45.3)	58 (41.7)	0.366	0.545
Follow-up at 6 months	58 (41.7)	74 (53.2)	3.693	0.055
**Continuous abstinence rates** (follow-up for 6 months)				
1-month continuous abstinence rate	44 (31.7)	71 (51.1)	10.812	0.001
3-month continuous abstinence rate	38 (27.3)	59 (42.4)	6.983	0.008

### Reasons for incomplete abstinence and factors influencing abstinence

In all, 181 (65.1%) smokers reached incomplete abstinence, comprising 101 cases (72.7%) in the abrupt cessation group and 80 cases (57.6%) in the gradual cessation group (χ^2^=6.983, p=0.008). After adjusting five predictive factors (i.e. FTND score, quitting motivation, exhaled CO at first visit, comorbidities and cessation methods), multiple logistic regression analyses (Table 3) showed that the abrupt cessation was a risk factor for successful smoking cessation than gradual cessation (AOR=2.39; 95% CI=1.15–3.85, p=0.013). In addition, compared to the ‘motivation of others’, the motivation of ‘prevention and treatment of own diseases’ reduced the risk of incomplete abstinence (AOR=0.87; 95% CI: 0.38–0.99, p=0.049), while there was no significant difference with the motivation by ‘Others’ (AOR=1.04; 95% CI: 0.45–2.06, p=0.279). No clear correlation was observed between other predictive factors (e.g. gender, age, age at smoking onset, number of cigarettes smoked daily, the number of years smoked, FTND score, marital status, employment status, level of education, underlying diseases, and the number of past quit attempts) and abstinence rates (p>0.5).

**Table 3:** Multiple logistic regression analyses of factors associated with incomplete abstinence (N=278)

*Variables*	*AOR (95% CI)^a^*	*p*
**Cessation methods**		
Gradual cessation (Ref.)	1	
Abrupt cessation	2.39 (1.15–3.85)	0.013
**Quitting motivation**		
Mobilization of others (Ref.)	1	
Prevention and treatment of own diseases	0.87 (0.38–0.99)	0.049
Other	1.04 (0.45–2.06)	0.279

AOR: adjusted odds ratio.

a The five predictive factors (i.e. FTND score, quitting motivation, exhaled CO at first visit, comorbidities and cessation methods) were adjusted.

In the present study, follow-up on smokers who reached incomplete abstinence indicated that the main reasons for cessation failure were (in the order of importance): inability to overcome tobacco addiction (98 cases, 54.1%), the need to relieve work stress by smoking (65 cases, 36.0%), social influence of other smokers (45 cases, 24.9%), lack of psychological preparedness and perseverance for smoking cessation (43 cases, 23.8%), stress and anxiety over the course of smoking cessation (36 cases, 19.9%), depression and unhappiness over the course of smoking cessation (29 cases, 16.0%), and other reasons (11 cases, 6.1%) (e.g. lack of confidence in cessation success, fear of post-abstinence weight gain, etc.) (Table 4).

**Table 4:** The list of reasons for cessation failures at 6-month follow-up by order of importance (multiple choices) (N=181)

*Reasons*	*Influence/severe influence n (%)*
Inability to overcome tobacco addiction	98 (54.1)
The need to relieve work stress by smoking	65 (36.0)
Social influence of other smokers	45 (24.9)
Lack of psychological preparedness and perseverance for smoking cessation	43 (23.8)
Stress and anxiety over the course of smoking cessation	36 (19.9)
Depression and unhappiness over the course of smoking cessation	29 (16.0)
Other reasons (e.g. lack of confidence in cessation success, fear of post-abstinence weight gain, etc.)	11 (6.1)

### Adverse events over the course of smoking cessation

Over the course of varenicline-aided cessation therapy, the incidence of adverse events in the abrupt cessation group was higher than that in the gradual cessation group. Nausea was the most common adverse event in the two groups with an incidence of 35.3% and 20.9%, respectively, and the incidence of insomnia being 18.7% and 7.2%, respectively. The differences between the abrupt cessation group and the gradual cessation group regarding the incidence of these two adverse events were statistically significant (χ^2^=7.128 and 8.169, p<0.05). As for the incidence of other adverse events, no statistically significant difference was observed between the two groups (p<0.05). Most adverse events started at Days 7–10 after drug administration with mild or moderate intensity (83.2%) with a median duration of 7 days. Moreover, no adverse event of severe intensity was reported (Table 5).

**Table 5:** Comparison of adverse events between the abrupt cessation group and the gradual cessation group (N=278)

*Adverse events^a^ (in >3% of all participants)*	*ALL (n=278) n (%)*	*Abrupt group (n=139) n (%)*	*Gradual group (n=139) n (%)*	*χ^2^*	*p*
Nausea	78 (28.1)	49 (35.3)	29 (20.9)	7.128	0.008
Dry mouth	32 (11.5)	19 (13.7)	13 (9.4)	1.271	0.260
Upper abdominal pain	15 (5.4)	8 (5.8)	7 (5.1)	0.070	0.791
Somnolence/fatigue	22 (7.9)	13 (9.4)	9 (6.5)	0.790	0.374
Abnormal dreams	26 (9.4)	15 (10.8)	11 (7.9)	0.679	0.410
Insomnia	36 (12.9)	26 (18.7)	10 (7.2)	8.169	0.004
Headache	13 (4.7)	7 (5.1)	6 (4.3)	0.081	0.776
Dizziness	17 (6.1)	10 (7.2)	7 (5.1)	0.564	0.453

a Coded using the Medical Dictionary for Regulatory Activities (MedDRA version 22.0).

## DISCUSSION

As a disease of addiction, tobacco dependence is an important global public health issue^16^. For smokers who experience moderate-to-severe nicotine dependence, the success rate of smoking cessation by willpower is relatively low and varenicline-aided smoking cessation therapy has been considered as an effective cessation method^17,18^. However, the course of smoking cessation needs to be personalized based on the evaluation results. Despite results of previous studies^3,5,7^ which showed that abrupt cessation therapy was a rapid-acting and effective cessation method, the gradual cessation therapy was also recommended^6^.

As a non-nicotinic partial agonist of the α4β2 nicotinic acetylcholine receptors, varenicline simulates the effect of nicotine in the reward center of the brain and competitively inhibits the receptor binding by nicotine delivered from cigarettes^19^. Systematic reviews/meta-analyses have confirmed that varenicline is the most effective single-form pharmacotherapy for smoking cessation^20-22^. In China, the recommended course of varenicline therapy (the most common medication for smoking cessation at present) is 3 months^12^, but in our clinical practice, most smokers who underwent pharmacotherapy generally achieved complete abstinence at about 4–8 weeks. Moreover, previous studies on abrupt cessation and gradual cessation were mostly based on efficacy observation in NRT-aided cessation programs, and the comparison between the two cessation methods in varenicline-aided cessation programs is insufficient. It is also unclear whether abrupt cessation is suitable for Chinese smokers who make active quit attempts. Existing literature reported^7^ higher abstinence rates in smokers who underwent abrupt cessation therapies than those who underwent gradual cessation therapies, but these findings are inconsistent with those of the present study. Gradual smoking cessation with pre-cessation varenicline therapy seemed to be more suitable for the cohorts we studied. No statistically significant difference was observed between the two groups regarding the short-term abstinence rates (7-day point prevalence abstinence rates), but as the follow-up periods were extended, the difference in abstinence rates between the two cessation methods became more significant. Thus, the gradual cessation therapy resulted in higher long-term abstinence rates. In addition, another study^23^ indicated that ‘Cue Restricted Smoking’ compared to the conventional ‘Target Quit Day’ method was associated with a significant increase in abstinence rates in smokers using varenicline. Cue Restricted Smoking is a treatment which involves gradual elimination of smoking through programmed restriction of the range of stimuli that lead to smoking.

We also performed further analyses of the factors that influence cessation success. Previous studies showed some common contributing factors, including health status, economic status, concomitant underlying diseases, and motivation to quit^24-26^. This study confirmed the previous observation that motivation to quit was a notable factor that influences cessation efficacy, but no correlation was found between cessation efficacy and other factors, such as concomitant underlying diseases and the degree of nicotine dependence. Furthermore, our research confirmed the choice of cessation methods (abrupt cessation and gradual cessation) was another important contributing factor. The results of this study demonstrated that gradual cessation therapies produced significantly higher long-term abstinence rates than abrupt cessation therapies did in varenicline-aided cessation treatment. Moreover, previous studies also indicated that the specific reasons for failures in smoking cessation were mostly poor self-control, difficulty in overcoming tobacco addiction, lack of effective cessation methods^27^, etc. At the 6-month follow-up of this study, we presented smokers who experienced cessation failures with multiple choice questions on the reasons for their cessation failure. One or more options were selected from the following reasons: inability to overcome tobacco addiction, the need to relieve work stress by smoking, social influence of other smokers, lack of psychological preparedness and perseverance for smoking cessation, etc. The smokers’ answers to the questions suggested that success in smoking cessation was influenced by tobacco addiction, work stress, and peer smokers. Therefore, smoking cessation services should be provided to correct the smokers’ social smoking behavior, help smokers overcome their psychological and physiological dependence on tobacco, and create an environment conducive to smoking cessation.

We are aware that smoking cessation treatment itself is accompanied by various withdrawal symptoms regardless of whether pharmacotherapies for smoking cessation were given or not. Therefore, in the study design and the analysis of the study results, we did not differentiate between the use of medication and nicotine withdrawal in terms of their correlation with adverse events (AEs). This study found that in oral varenicline-aided cessation treatment, the incidence of AEs in the gradual cessation group was lower than that in the abrupt cessation group. This phenomenon could be due to the fact that abrupt cessation could cause an abrupt drop in the level of nicotine and consequently exacerbate the withdrawal symptoms. In contrast, milder withdrawal symptoms and lower incidence of AEs were observed in the gradual cessation group due to a gradual and steady decrease in the level of nicotine. Moreover, this study showed that the three most frequent AEs were nausea, insomnia, and dry mouth, with higher incidence rates than those of other treatment-related AEs. The incidence rate of AEs in the gradual cessation group was similar to the findings of previous studies^18,28^, but the abrupt cessation group seemed to have a higher incidence of AEs. Moreover, these AEs were mostly of mild or moderate intensity, with a relatively short duration (median duration: 7 days). In summary, gradual cessation was safer than abrupt cessation in the varenicline-aided treatment of smokers who experienced moderate-to-severe nicotine dependence. In addition, another study had found that varenicline could prolong ventricular repolarization parameters and might induce arrhythmias after smoking cessation^29^. Therefore, varenicline-related adverse reactions require further identification and study.

### Limitations

Given the small sample size, few follow-up times, single biological evaluation and the short follow-up time of this single-center retrospective study, the study has limitations. Therefore, further large-sample, more follow-up times, multiple biological evaluations, multi-center, long-term, prospective randomized controlled clinical studies are needed for in-depth analyses and discussions.

## CONCLUSIONS

The study demonstrated that gradual smoking cessation with pre-cessation varenicline therapy produced higher continuous abstinence rates and relatively milder withdrawal symptoms than abrupt cessation.

## Data Availability

The data supporting this research are available from the authors on reasonable request.
